# Review of "*Hemoparasites of the Reptilia. Color Atlas and Text*" by Sam R. Telford, Jr

**DOI:** 10.1186/1756-3305-2-40

**Published:** 2009-09-02

**Authors:** George Poinar

**Affiliations:** 1 Department of Zoology, 3029 Cordley Hall, Oregon State University, Corvallis, Oregon 97331-2914, USA

## Abstract

Book review of *"Hemoparasites of the Reptilia. Color Atlas and Text" *by Sam R. Telford, Jr.

## Review

This work presents a fascinating and detailed view of blood parasites (hemoparasites) of reptiles. As indicated by the author, the aim of this work is to compile all of the published data on the morphology of reptilian unicellular parasites. This is supplemented by unpublished data acquired by the author, his students and colleagues during the past 45 years. Included are morphometric descriptions of species of plasmodiids, hemogregarines, hemococcidians, trypanosomatids, piroplasmorids and miscellaneous blood parasites from lizards, snakes, turtles, crocodilians and even the tuatara. Over half of the text deals with malarial parasites of the genera *Plasmodium*, *Haemocystidium *and *Saurocytozoon*.

The species descriptions are easy to follow since they are standardized under the headings of diagnosis, hosts, geographic distribution, prevalence, morphological variation, sporogony and effects on the host. The criteria for species determination appear to be based mainly on the morphology of the various parasite stages and their position within the host cells. In many cases, differences are obvious in the accompanying color plates, while in others, the degree of variation appears to overlap with that of closely related species. The ability to distinguish between the developmental stages of these parasites certainly comes with experience.

While one might be tempted to use host records to identify hemoparasites, it is preferable to also examine morphological characters when attempting a specific determination.

General sections in the book are separated by host and geographical range (e.g. *Plasmodium *species of Neotropical lizards, etc.), which allows researchers to turn to a particular locality in determining what type of infections occur there or what they may have discovered.

Some host-parasite associations included in this work are surprising. Parasitologists have been stating for years that crocodilians do not get malaria, however, included in the present work is a plasmodiid parasite of Neotropical crocodilians that apparently has gone largely unnoticed for the past decade. Since sylvatic crocodilians are not the easiest animals to test for blood parasites, the absence of earlier reports may have been due to the limited number of samples taken. The problem of obtaining blood from dangerous hosts may also explain why there are no blood parasites reported for the Komodo dragon, although infections occur in other species of *Varanus*.

There is a useful appendix dealing with the identification of reptilian hemoparasites and excellent color photographs demonstrate the various groups covered in the body of the work. Non-specialists might have found it helpful if the author had added a key to the various genera or at least to the different groups of blood parasites to assist the identification process.

While reading the numerous species descriptions, it is apparent that little information has been acquired about the sporogonic stages of these parasites. It is unfortunate that so many of the vectors are unknown and many of those that are listed are based on laboratory infections, which may or may not indicate the natural vectors. This area is clearly wide open for future generations.

A special section summarizing pathological effects of the various hemoparasites on the survival and well-being of the hosts would have been useful, as well as the author's thoughts on the evolutionary history of these parasites.

"Hemoparasites of the Reptilia" demonstrates the amazing diversity of reptilian blood parasites and certainly is a "must have" for those studying blood parasites of reptiles or any other vertebrate group for that matter. I highly recommend it for general protozoologists, parasitologists and vertebrate ecologists as well. Telford has provided a detailed account of rare parasites that can now be appreciated by all.

## Competing interests

The author declares that they have no competing interests.

